# Comparative Evaluation of PRISM III, PIM 2, and PELOD Scores as Predictors of Outcome in Pediatric Intensive Care Unit Patients: An Experience From a Single Center in South Gujarat, India

**DOI:** 10.7759/cureus.104420

**Published:** 2026-02-27

**Authors:** Dorothy Sengupta, Shreya Bhatt, Swati Patel, Poonam Singh

**Affiliations:** 1 Pediatrics, Surat Municipal Institute of Medical Education and Research (SMIMER), Surat, IND; 2 Pediatrics, Anand Hospital, Surat, IND; 3 Community Medicine, Surat Municipal Institute of Medical Education and Research (SMIMER), Surat, IND

**Keywords:** mortality predictors, pelod, picu, pim 2, prism iii

## Abstract

Aim: To assess and compare Pediatric Risk of Mortality III (PRISM III), Pediatric Index of Mortality 2 (PIM 2), and Pediatric Logistic Organ Dysfunction (PELOD) scores for predicting mortality in a pediatric intensive care unit (PICU) setting.

Design and setting: A cross-sectional, hospital-based, analytical observational study conducted in an eight-bed PICU.

Methods: PICU patients between the ages of 1 month and 17 years fulfilling the inclusion criteria were included in this study. PIM 2 score was calculated within the first hour of admission; PRISM III and PELOD scores were calculated at 24 hours. Outcomes of survival or non-survival were recorded. Scoring system performance was assessed using indices of discrimination and calibration. Predictive accuracy for mortality was evaluated with receiver operating characteristic (ROC) curve analysis, and calibration between predicted and observed mortality was examined using the Hosmer-Lemeshow goodness-of-fit test.

Results: Data from 138 patients were analyzed (M: F=0.8:1) with a mean age of 62.5 months and a median of 24 months. Respiratory ailments were the highest (46.4%), followed by central nervous system diseases (18.1%), and overall mortality was 20.3%. Area under the ROC curve was 0.984 for PRISM III, 0.836 for PIM 2, and 0.872 for PELOD. PRISM III was a better predictor of mortality risk, followed by PIM 2 and PELOD. The goodness-of-fit test showed good calibration for all three scores, but PRISM III had the better power compared to PIM 2 and PELOD.

Conclusion: In our PICU setting, the PRISM III score was a better predictor of mortality risk, and the performance of PELOD was the least.

## Introduction

Scoring systems allow the estimation of patient mortality risk in the intensive care unit (ICU) by assigning a weighted score to the patient and predicting the outcome [1]. Patient mortality is affected not only by ICU performance but also depends on other factors such as demographic and clinical characteristics of the population, infrastructure, and non-medical factors, case mix, and admission practices [2]. Scores were developed and extensively validated in their own settings for these purposes. Indian population and conditions are different from those in developed countries in terms of case mix, demographic characteristics, admission practices, and non-medical factors; hence, there is a need for field testing of these scoring systems in Indian settings.

The lack of consistency, reliability, and accuracy in physicians' subjective opinions concerning patient status necessitates quantitative clinical scores in the paediatric intensive care unit (PICU) setting. Over the past three decades, multiple scoring systems have been developed for ICU patients. These systems enable objective assessment of disease severity and provide estimates of in-hospital mortality. The prediction is derived from routinely collected patient data, with each variable assigned a specific weight; the aggregate of these weighted values constitutes the severity score [1].

Scoring systems like the Paediatric Risk of Mortality (PRISM) and Paediatric Index of Mortality (PIM) are widely used. They facilitate objective assessment of illness severity and enable mortality risk adjustment in heterogeneous patient populations, with conversion of clinical variables into a quantified probability of death through logistic regression models [3].

The capacity to reliably predict a patient's risk of mortality is critical because it would serve many different purposes, such as assessing patients’ prognosis, monitoring ICU performance, optimizing ICU resource allocation, evaluating therapy success, and adequately matching the severity of illness in clinical research [4,5]. The scoring systems provide objective measures for inter- and intra-unit comparisons with time and also provide useful information for comparing the severity of illness of patients at the time of enrolment into clinical trials [6,7].

Data from developing nations have conflicting results, like under-prediction of mortality, poor sensitivity, and different calibrating and discriminative abilities [8]. We planned this study in our PICU using three commonly used scores [9,10,11,12]: PRISM III [6,7], PIM 2 [13], and Paediatric Logistic Organ Dysfunction (PELOD) [14,15] to assess which score has better predictive value for mortality. PRISM III was chosen for its ease of evaluation [7], PIM 2, as it avoids the early treatment bias [13], and PELOD as a descriptive score [14]. We calculated PELOD at only 24 hours for comparison with other scores.

## Materials and methods

Study design, setting, and duration

This was a cross-sectional, single-centre, analytical observational study conducted in an eight-bed tertiary care PICU of the Surat Municipal Institute of Medical Education and Research (SMIMER), Surat, Gujarat, India, from September 2017 to August 2018.

Inclusion and exclusion criteria

The exclusion and inclusion criteria are detailed in Table 1. 

**Table 1:** Inclusion and Exclusion Criteria

Category	Criteria
Inclusion	Patients admitted to the PICU within the first hour of visiting the hospital
	Age: 1 month to 204 months (17 years)
	Informed consent obtained
Exclusion	Patients who expired within the first 24 hours
	Patients whose scores were not evaluated at admission and at 24 hours
	Patients transferred to other hospitals within 24 hours
	Non-availability of investigations for scoring (incomplete data)
	Patients not providing consent
Other considerations	Re-admissions attributable to different diagnoses were classified as new admissions
	All patients were treated as per standard protocols by residents and consultants

Data collection

Data for each enrolled patient were collected within 24 hours of admission using a structured proforma that included demographic, clinical, laboratory, and outcome variables required for estimation of PRISM III, PIM 2, and PELOD scores. Physiologic variables where normal values change with age were stratified by age (neonate (0-1 month), infant (>1 to 12 months), child (>12 to 144 months), adolescent (>144 months)). For variables measured multiple times within the first 24 hours, the most abnormal (worst) values were recorded, and scores were calculated.

Score calculation

PRISM III

The total PRISM III score is calculated at 24 hours, as the sum of four components: (i) cardiovascular and neurologic sub-scores, (ii) acid-base and blood gas sub-scores, (iii) chemistry sub-scores, and (iv) haematology sub-scores. The overall score ranges from 0 to 74 (Table 2) [7]. 

**Table 2:** PRISM III Scoring System: Variables and Weighting Pollack et al. [7] BUN, blood urea nitrogen; PT / PTT, prothrombin time / partial thromboplastin time PaO_2_: arterial partial pressure of oxygen; PaCO_2_, arterial partial pressure of carbon dioxide

Domain	Variable	Range / Criteria	Score Weight
Cardiovascular	Systolic blood pressure	Age-specific thresholds	0–7
	Heart rate	Age-specific thresholds	0–4
	Temperature	<33°C or >40°C	0–3
Neurology	Pupillary reactions	Fixed or dilated pupils	0–11
	Glasgow Coma Scale	≤ 8	0–5
Acid–Base / Blood Gas	pH	<7.28 or >7.55	0–6
	PaO₂	<50 mmHg	0–7
	PaCO₂	>65 mmHg	0–5
	Total CO₂ / HCO₃	<15 mmol/L	0–5
Chemistry	Glucose	<40 or >300 mg/dL	0–5
	Potassium	<2.5 or >6.5 mmol/L	0–5
	Creatinine (age-specific thresholds)	Elevated	0–7
	BUN	>20 mg/dL	0–5
Hematology	White blood cell count	<3,000 or >40,000 /mm³	0–4
	Platelet count	<50,000 /mm³	0–5
	PT / PTT	>1.5 × control	0–5
Total score		Sum of all sub-scores	0–74

PIM Scores

PIM 2 [13] scores, based on 11 physiologic variables, were calculated within an hour of admission using the online PIM 2 calculator (https://qxmd.com) using the following formulae: 

\( \begin{aligned} PIM2val &= (0.01395 \times |SBP - 120|) \\ &+ (3.0791 \times Pupils) \\ &+ \left(0.2888 \times \left(\frac{100 \times FiO_2}{PaO_2}\right)\right) \\ &+ (0.1040 \times |Base\ Excess|) \\ &+ (1.3352 \times MechVent) \\ &- (0.9282 \times Elective) \\ &- (1.0244 \times Recovery) \\ &+ (0.7507 \times Bypass) \\ &+ (1.6829 \times HRdiag) \\ &- (1.5770 \times LRdiag) \\ &- 4.8841 \end{aligned} \) Risk of death: ${PIM2} = \frac{e^{\mathrm{PIM2val}}}{1 + e^{\mathrm{PIM2val}}}$ Score range : 0-50.999

PELOD Scores

PELOD scores [14], based on 12 physiologic variables, were calculated at 24 hours. The total PELOD score is the sum of neurological, cardiovascular, renal, respiratory, haematological, and hepatic subscores. The score range is 0-40 (Table 3). 

**Table 3:** Paediatric Logistic Organ Dysfunction (PELOD) Scoring System PELOD is calculated at 24 hours after PICU admission, and only the most adverse value for each variable within 24 hours is used [14]. * Either of the two values can be used. NA, not applicable; SBP, systolic blood pressure; PaO_2_/FiO_2_: arterial partial pressure of oxygen/fraction of inspired oxygen; PaCO_2_, arterial partial pressure of carbon dioxide; INR, international normalized ratio

Organ dysfunction and variable	Scoring system			
	0	1	10	20
Neurological				
Glasgow Coma Scale	12 to 15	7 to11	4 to 6	3
Pupillary reaction	Both reactive	NA	Both fixed	NA
Cardiovascular				
Heart rate (beats/min)				
<12 years	=195	NA	>195	NA
12 years	=150	NA	>150	NA
And/or SBP (mmHg)				
<1 month	>65	NA	35-65	<35
1 month -1 year	>75	NA	35-75	<35
1-12 years	>85	NA	45-85	<45
12 years	>95	NA	55-95	<55
Renal				
Creatinine (µmol/L)				
<7 days	<140	NA	140	NA
7 days-1 year	<55	NA	55	NA
1-12 years	<100	NA	100	NA
12 years	<140	NA	140	NA
Respiratory				
PaO_2_ (mmHg)/FiO_2_	>70	NA	=70	NA
PaCO_2_ (mmHg)	=90	NA	>90	NA
Mechanical ventilation	No ventilation	Ventilation	NA	NA
Haematological				
White blood cell count (10^9/L)	≥4.5 and	1.5-4.4	<15	NA
Platelets (10^9/L)	≥35	<35	NA	NA
Hepatic				
Aspartate transaminase (IU/L)	<950 and	≥950 or	NA	NA
Prothrombin time (or INR)*	<60 (<1.40)	≥60 (≥1.40)	NA	NA

Statistical analysis

All analyses were performed using Statistical Package for the Social Sciences (SPSS), version 22.0 (IBM Corp., Armonk, NY). Categorical variables were summarized as frequencies and percentages. The Kolmogorov-Smirnov test was used to assess the normality of data distribution. Continuous variables - not normally distributed were expressed as median (interquartile range, IQR). Independent group comparisons were performed using the non-parametric Mann-Whitney U test. Binary and categorical data were compared using the χ² test, Fisher’s exact test, or Yates’ correction, as appropriate. 

Model discrimination, defined as the ability to distinguish survivors from non-survivors, was evaluated using the area under the receiver operating characteristic curve (AUC-ROC) with corresponding 95% confidence intervals (CIs). Sensitivity, specificity, and optimal cut-off thresholds were determined; the best cut-off was identified as the value maximizing the Youden index (Sensitivity + Specificity -1). Discrimination was classified as acceptable (AUC 0.70-0.79), good (AUC ≥0.80), or excellent (AUC ≥0.90) [16-18]. Areas under the ROC curves (AUCs) were compared using a Z-test for correlated ROC curves [19]. The calculated Z value (Zcal) represents the standardized difference between two AUCs relative to the standard error of their difference. A p-value < 0.05 was considered statistically significant. 

Calibration refers to the degree of agreement between predicted mortality probabilities generated by a prognostic model and the actual observed outcomes across the spectrum of risk. It evaluates whether the model systematically overestimates or underestimates risk in a given population. Model calibration was assessed using the Hosmer-Lemeshow goodness-of-fit test. Based on predicted mortality probabilities, patients were ranked and stratified into 10 approximately equal-sized risk groups (deciles of risk). Within each group, the observed number of deaths was compared with the number expected according to the model [17].

The Hosmer-Lemeshow test statistic was calculated by summing the squared differences between observed and expected events across all risk groups, weighted by the variance of the expected values. A non-significant p-value (>0.05) indicated good calibration, implying close agreement between predicted and observed mortality across risk strata. Conversely, a significant p-value (<0.05) suggested poor calibration, reflecting systematic deviation between predicted and observed outcomes and limited applicability of the model to the study population.

To complement the Hosmer-Lemeshow test and provide a quantitative measure of the magnitude of miscalibration, effect size was expressed using Cohen’s w. Cohen’s w quantifies the degree of deviation between observed and expected outcomes, independent of sample size, and was interpreted as small (0.10), medium (0.30), or large (0.50) miscalibration. This combined approach allowed for both statistical and clinical interpretation of model calibration.

The ultimate goal of a prognostic model is to provide reliable, transportable tools that can be feasibly implemented in clinical practice. In resource-constrained PICU, especially in developing countries, a balance between simplicity and predictive performance is crucial [17]. Both complex and simplified scoring systems can aid clinicians in prioritizing care for critically ill children [17]. AUC values ≥0.90 are considered to reflect excellent discrimination, with values approaching 1.0 indicating superior predictive accuracy [16,18].

## Results

A total of 305 children were admitted to the PICU during the study period. Of these, 138 patients met the inclusion criteria and were analyzed, while 167 patients were excluded as per the predefined criteria. Among the study cohort, 74 (53.6%) were female. The median age was 24 months (IQR: 7-132). Respiratory diseases constituted the leading cause of PICU admission (64 patients (46.4%)), followed by central nervous system disorders (25 (18.1%)), cardiovascular diseases (20 (14.5%)), hematological disorders (11 (8.0%)), hepatobiliary diseases (7 (5.1%)), abdominal conditions (6 (4.3%)), and renal disorders (5 (3.6%)). Mechanical ventilation was required in 43.1% of patients. The median length of PICU stay was 5 days (IQR: 4.5-9). Based on outcomes, patients were divided into a survival group (110 (79.7%)) and a non-survival group (28 (20.3%)). No significant differences were observed between survivors and non-survivors with respect to age, gender, admission diagnosis, or length of PICU stay (p > 0.05) (Table 4).

**Table 4:** Comparison of demographic and clinical data of the two groups df, degree of freedom; OR, odds ratio; NA, not applicable

Characteristics		Survival group; n (%), n=110	Non-survival group; n (%), n=28	P-value	df, OR
Gender	Male	52 (47.3)	12 (42.9)	0.6757	df=1, OR=1.195
	Female	58 (52.7)	16 (57.1)		
Median age (months)		16 (6-132)	24 (9-147)	0.198	
Age group	1 month to 12 months	37 (33.6)	11 (39.3)	0.5752	df=1, OR=0.7847
	> 1 year to ≤ 12 years	42 (38.2)	8 (28.6)	0.3449	df=1, OR=1.544
	>12 years to ≤17 years	31 (28.2)	9 (32.1)	0.9674	df=1, OR=0.8284
Diagnosis	Respiratory system	54 (49)	10 (35.7)	0.2054	df=1, OR=1.736
	Central nervous system	19 (17.3)	6 (21.4)	0.6102	df=1, OR=0.7656
	Cardiovascular system	14 (12.7)	6 (21.4)	0.2436	df=1, OR=0.5347
	Haematological system	8 (7.3)	3 (10.7)	0.834	df=1, OR=0.6535
	Hepatobiliary system	5 (4.5)	2 (7.1)	0.9387	df=1, OR=0.085
	Abdominal system	6 (5.5)	0 (0)	0.4986	df=1, OR=NA
	Renal system	4 (3.6)	1 (3.6)	>0.99	df=1, OR=0.9474
Hospital stay (days)		5 (7-8)	7.5 (5-12)	0.06	

All three severity scores: PRISM III, PIM 2, and PELOD, were significantly higher in the non-survival group compared with survivors (p < 0.001), indicating strong discriminatory ability between outcome groups. The clear separation of median values and interquartile ranges suggests clinically useful score thresholds that may aid early risk stratification in the PICU (Table 5).

**Table 5:** Comparison of severity scores between survival and non-survival groups. PRISM III, Pediatric Risk of Mortality III; PIM 2, Pediatric Index of Mortality 2; PELOD, Pediatric Logistic Organ Dysfunction; IQR, interquartile range

Severity scores	Survival group (n=110)	Non-survival group (n=28)	P-value
PRISM III (median, IQR)	5.50 (3-9)	26 (24-32)	<0.001
PIM 2 (median, IQR)	8.90 (6.34-13.89)	22.64 (15.29-31.66)	<0.001
PELOD (median, IQR)	10 (1-12)	22 (12-31)	<0.001

The observed mortality in the cohort was 20.3% (28 deaths), while predicted mortality was 19.6% (27 deaths) by PRISM III, 11.6% (16 deaths) by PIM 2, and 15.2% (21 deaths) by PELOD. Among the three models, PRISM III showed the best calibration between predicted and observed outcomes (w ≈ 0.20, small effect), whereas PIM 2 (w ≈ 0.30) and PELOD (w ≈ 0.29) indicated moderate miscalibration (medium effect). Overall, smaller effect sizes reflected better calibration, confirming PRISM III as the most reliable model in this cohort (Tables 6-7).

**Table 6:** Performance values of PRISM III, PIM 2 and PELOD for predicting mortality. PRISM III, Pediatric Risk of Mortality III; PIM 2, Pediatric Index of Mortality 2; PELOD, Pediatric Logistic Organ Dysfunction; AUC, area under the curve; df, degree of freedom; ES, effect size

Scores	Hosmer-Lemeshow goodness-of-fit test	AUC	P-value	Cut-off score (Youden Index)	Sensitivity	Specificity	Accuracy
		(95% CI)					
PRISM III	χ^2^ = 5.33, df=1, ES=0.204	0.984	<0.0001	14.5	91.80%	96.40%	92.80%
	p=0.722	(0.964-1.0)					
PIM 2	χ^2^ =11.15, df=1, ES=0.295	0.836	<0.0001	17.44	75%	88.20%	85.50%
	p=0.193	(0.745-0.928)					
PELOD	χ^2^ =10.43, df=1, ES=0.285	0.872	<0.0001	11.5	70%	85.70%	73.20%
	p=0.165	(0.809-0.935)					

**Table 7:** Hosmer-Lemeshow χ² statistics and decile calibration of the severity scores PRISM III, Pediatric Risk of Mortality III; PIM 2, Pediatric Index of Mortality 2; PELOD, Pediatric Logistic Organ Dysfunction

Predicted probability	Non-survival group		Survival group		Total
	Observed	Expected	Observed	Expected	
PRISM III					
0-0.532	13	13.781	1	0.219	14
0.533-0.602	12	10.473	1	2.527	13
0.603-0.884	2	2.85	11	10.15	13
0.885-0.966	1	0.476	11	11.524	12
0.967-0.978	0	0.2	14	13.8	14
0.979-0.982	0	0.109	17	16.891	17
0.983-0.991	0	0.065	19	18.935	19
0.992-0.994	0	0.024	12	11.976	12
0.994-0.997	0	0.013	10	9.987	10
0.998-1	0	0.009	14	13.991	14
PIM 2					
0-0.415	11	10.73	3	3.27	14
0.414-0.651	6	6.021	8	7.979	14
0.652-0.810	4	3.431	10	10.569	14
0.811-0.866	2	2.207	12	11.793	14
0.867-0.908	0	1.541	14	12.459	14
0.909-0.922	2	1.153	12	12.847	14
0.923-936	0	1.001	14	12.999	14
0.937-0.946	3	0.807	11	13.193	14
0.947-0.956	0	0.684	14	13.316	14
0.967-0.973	0	0.425	12	11.575	12
PELOD					
0-0.428	12	12.08	4	3.92	16
0.429-0.760	4	5.96	9	7.04	13
0.761-0.820	2	2.021	8	7.979	10
0.821-0.845	6	2.779	12	15.221	18
0.846-0.867	3	1.322	7	8.678	10
0.868-0.887	1	2.707	23	21.293	24
0.888-0.970	0	0.191	5	4.809	5
0.971-0.975	0	0.511	21	20.489	21
0.975-0.979	0	0.429	21	20.571	21

As per the Youden Index, in our cohort, PRISM III demonstrated the strongest overall performance, with an optimal cut-off score of 14.5, yielding excellent sensitivity (91.8%) and specificity (96.4%), and an overall diagnostic accuracy of 92.8%. This indicates that PRISM III is highly effective in correctly identifying both survivors and non-survivors, making it a reliable tool for early risk stratification in the PICU.

For PIM 2, the optimal cut-off score was 17.44, which achieved a sensitivity of 75% and specificity of 88.2%, with an overall accuracy of 85.5%. Although slightly less sensitive than PRISM III, PIM 2 demonstrated good specificity, suggesting utility in identifying patients at higher risk of mortality while minimizing false-positive predictions.

PELOD showed an optimal cut-off score of 11.5, with sensitivity and specificity of 70% and 85.7%, respectively, and an accuracy of 73.2%. This reflects moderate discriminatory performance, indicating that PELOD may be more useful as a marker of evolving organ dysfunction rather than as a standalone mortality prediction tool (Table 6).

Importantly, all three scoring systems demonstrated good calibration on Hosmer-Lemeshow testing with small-to-moderate effect sizes, supporting the validity of these Youden Index-derived thresholds in our population. Collectively, these findings suggest that while PRISM III offers the best balance between sensitivity and specificity, PIM 2 and PELOD remain valuable adjuncts for mortality risk assessment and clinical decision-making in the PICU.

**Figure 1:**
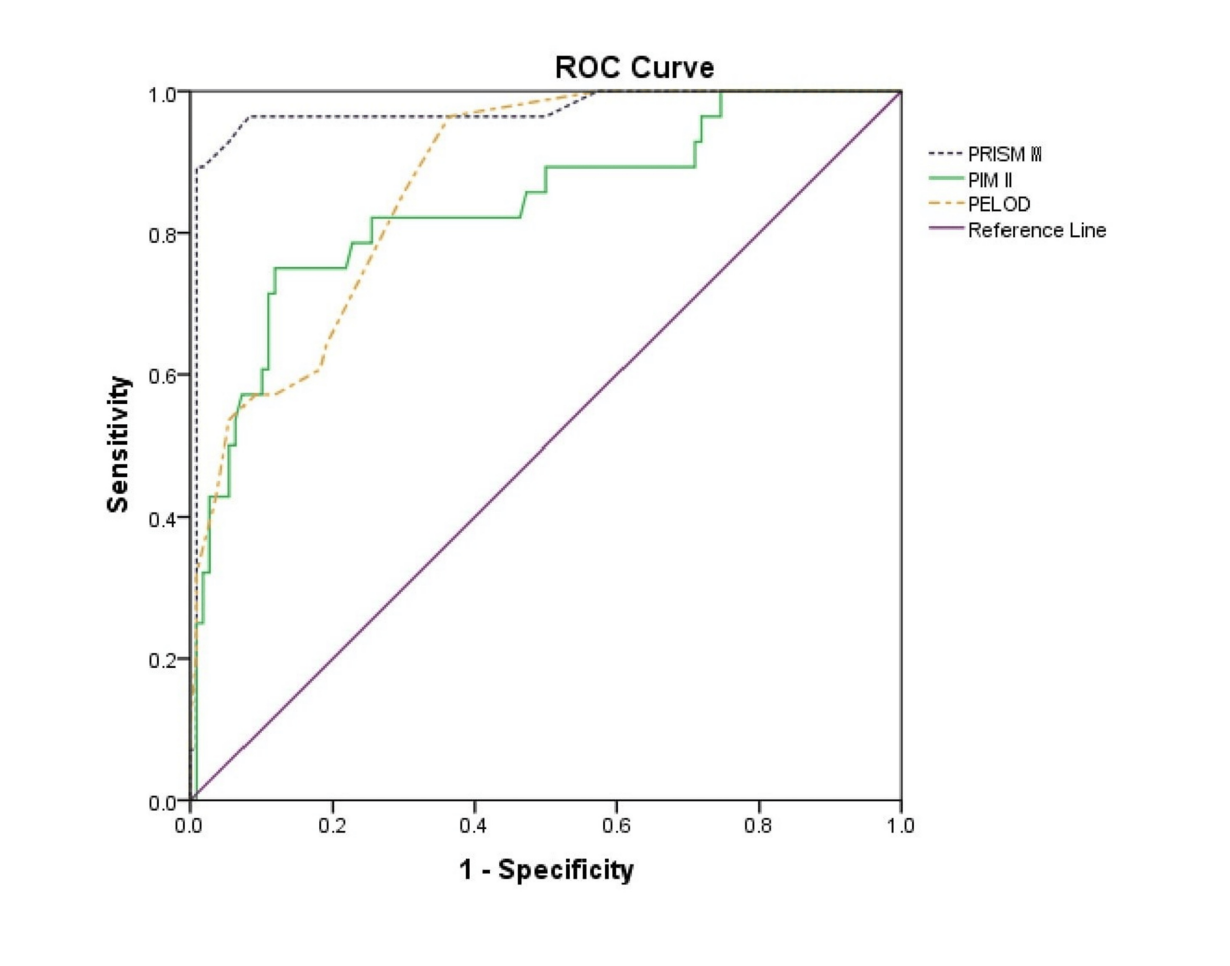
ROC for predicting survival outcomes based on PRISM III, PIM 2 and PELOD scores PRISM III, Pediatric Risk of Mortality III; PIM 2, Pediatric Index of Mortality 2; PELOD, Pediatric Logistic Organ Dysfunction

Analysing the AUC showed that the power of the prediction model was outstanding for PRISM III (AUC = 0.984), and was very good for PIM 2 (AUC = 0.836) and PELOD (AUC = 0.872) (Figure 1).

ROC curve analysis demonstrated that PRISM III had significantly greater discriminatory ability compared with PIM 2 and PELOD. The AUC of PRISM III was significantly higher than that of PIM 2 (Zcal = 3.08, p = 0.0021) and PELOD (Zcal = 3.34, p = 0.0008). 

## Discussion

Mortality prediction models serve as important and valuable tools for evaluating the quality of care delivered to critically ill patients. Children admitted to PICU have a high risk of mortality due to multiorgan failure; hence, appropriate tests to predict mortality are useful for effective and immediate care [7]. Our findings compare the prediction of mortality by using three scores, PRISM III, PIM 2, and PELOD, in the pediatric intensive care unit at a teaching tertiary care hospital. The discrimination and calibration of these scores were assessed and compared. 

In our study, age and gender had no significant influence on outcome and mortality, which is comparable to the observations noted by another Indian study [18]. We observed that the predicted mortality by PRISM III (19.6%) was closest to the observed mortality of 20.3%, whereas PIM 2 (11.6% ) and PELOD (15.2%) underestimated mortality.

The discriminatory power of the three scoring systems was evaluated using the area under the receiver operating characteristic curve (AUC). PRISM III demonstrated excellent discrimination with an AUC of 0.984, followed by PELOD (0.872) and PIM 2 (0.836). These findings indicate that all three models were able to distinguish survivors from non-survivors, consistent with observations from previous studies [12,16,20]. When considering PICU mortality as an outcome, the PRISM III demonstrated superior discriminative ability.

All three scoring systems demonstrated good calibration in our cohort, reflecting a close agreement between predicted and observed mortality across the spectrum of illness severity. This was evidenced by non-significant Hosmer-Lemeshow goodness-of-fit χ² statistics for all models, indicating the absence of systematic overestimation or underestimation of mortality risk within the deciles of predicted probability. Among the three, PRISM III showed the lowest χ² value (χ² = 5.330, p = 0.722), suggesting the most consistent alignment between expected and observed outcomes, while PIM 2 (χ² = 11.15, p = 0.193) and PELOD (χ² = 10.434, p = 0.165) also demonstrated acceptable calibration.

The favorable calibration observed across all models suggests that these scoring systems are applicable to our patient population despite differences in case mix, disease severity, and healthcare delivery compared with the settings in which the models were originally developed. Importantly, the high p-values observed, particularly for PRISM III, indicate minimal deviation between predicted and observed mortality rates across risk strata.

Therefore, calibration findings were interpreted alongside effect size measures and discrimination metrics to provide a more comprehensive assessment of model performance. While good calibration supports the reliability of predicted mortality estimates, it does not alone guarantee optimal prognostic performance, underscoring the importance of evaluating both calibration and discrimination when validating severity-of-illness scoring systems in local populations.

However, some studies have reported poor calibration of these scoring systems. The calibration results should be interpreted cautiously, as the Hosmer-Lemeshow test is sensitive to sample size and may fail to detect clinically relevant miscalibration in smaller cohorts, and larger cohorts tend to increase the likelihood of rejecting the null hypothesis of agreement between predicted and observed outcomes, and to the choice of cut-off points. Variability in patient profiles, pre-hospital care, delayed referrals, available resources, and PICU caseloads may further contribute to differences in the performance of the three evaluated scores [17].

In terms of underlying disease, the respiratory system was most frequently affected at admission (46.4%), which is consistent with findings of other studies [16,20,21]. Mortality was also higher for respiratory diseases, 35.7%, aligning with another study [16]. The overall observed mortality in our cohort was 20.3%, comparable with Costa et al. (15%) [22] and Qureshi et al. (28.7%) [12]. The PICU mortality observed in our study is higher than in European and US PICUs (12.4% vs 2.5%) [23,24], but is within the mortality range reported from developing countries: 8.4% in Korea [25] to 40% in Egypt [21]. Variations in mortality may be attributed to differences in disease profiles, standards of care, availability of equipment, and overall health system development, as well as differences in the severity of illness among admitted patients [26].

PIM 2 is assessed at admission, has fewer variables, and does not account for disease progression and multi-organ dysfunction, whereas PELOD, evaluated at 24 hours, incorporates evolving organ dysfunction, explaining its better prediction value in our setting.

In one of the previous studies from India, the observed mortality was 18 (17.6%), and both PRISM III and PIM 2 underestimated the mortality; the predictive mortality being 50% and 38.9%, respectively [20]. Two other studies also showed the underprediction of mortality with the PRISM III score as compared to the PIM 2 and PELOD score [27,28]. The reason is the subjective error in the calculation of scores. 

In our analysis, the AUC of PRISM III was significantly higher than PIM 2 (Zcal = 3.08, P = 0.0021) and PELOD (Zcal = 3.34, P = 0.0008), which is comparable to other studies [12,17]. Costa et al. [22] found the PRISM III score to have good discrimination, and therefore, it can be a useful tool for the assessment of prognosis for patients admitted to the PICU. Conversely, two studies reported poor discrimination (AUC = 0.667 [23] and 0.56 [29]), possibly reflecting study-specific factors such as inclusion of children on extracorporeal support. This highlights that prediction of short-term outcomes may be more reliable than longer-term prognostication.

A cohort study done in the United States demonstrated that higher PRISM III scores were associated with increased mortality, indicating that PRISM III adequately predicts the risk of non-survival in critically ill pediatric patients [16]. Several other studies have similarly confirmed the good predictive performance of PRISM III [16,30,31], consistent with our findings.

Thukral et al. [18] studied PELOD and found the AUC to be 0.80, which is comparable to our study, but Qureshi et al. [12] found the AUC for PELOD to be 0.77, which is a bit lower than our study. Patki et al. [8] found that PIM 2 had a better power of calibration than PRISM III, which is also supported by Qureshi et al. [12]. PIM 2 has an edge over PRISM III as it has few variables and is estimated in the first hour of admission as opposed to the 24 hours of PRISM. Early evaluation in PIM 2 also allows for early and effective treatment commencement. Comorbid conditions, as well as diagnosis at admission to the PICU, invariably affect the outcome of the patients.

Limitations of the study

The use of these severity scores imposes a monetary burden on guardians due to the investigations required, making them challenging to implement in resource-limited settings. Furthermore, our PICU caters exclusively to medical patients, so we did not include surgical patients. The observed discrimination and calibration of the severity scores should be interpreted with caution, as potential survivorship bias may have influenced model performance.

Strength of this study

A good comparison of all three scores was possible. The data were thoroughly checked for accuracy and reliability, employing robust statistical methods to validate the results. The outcome measures were clearly defined and clinically relevant, thereby contributing to the external validity and real-world applicability of this study. The methodology used is easily reproducible, and our results agree with those of previous studies. This will add data to the present scenario for Indian PICU setups and may be helpful for further research.

Implications and recommendations

Despite the small sample size, PRISM III and PIM 2 are recommended for early mortality risk assessment in PICU patients. All three scores were significantly higher in non-survivors (p < 0.001), with clear separation of median scores and interquartile ranges, suggesting clinically meaningful thresholds. In our cohort, PRISM III ≥20, PIM 2 ≥15, and PELOD ≥20 were associated with higher mortality, while lower scores (PRISM III <10, PIM 2 <10, PELOD <10) indicated a favorable prognosis. Intermediate scores warrant close monitoring.

These thresholds should guide early risk stratification, triage, and resource allocation rather than serve as definitive prognostic cut-offs. Prospective validation in larger Indian cohorts using AUROC-based optimization is recommended. Future studies incorporating cost-effectiveness analysis, time-to-event analysis, broader case mixes, and longer follow-up may further refine mortality prediction.

## Conclusions

Certain conditions may generate disproportionately high severity scores without corresponding increases in mortality. Thus, predicted mortality from a given score reflects group-level risk rather than individual patient outcomes. In our study, PRISM III demonstrated superior predictive performance compared with PIM 2 and PELOD in terms of discrimination, calibration, and accuracy. However, PIM 2, which is calculated at admission and requires fewer investigations, may be more practical in resource-limited settings despite slightly lower predictive performance. Given the similarity of patient profiles and healthcare challenges across many developing countries, these findings may aid in the rapid evaluation and management of critically ill children. Larger, well-designed multicentric studies are warranted to further validate the performance of these models in such settings.
